# Work Stress and the Development of Chronic Diseases

**DOI:** 10.3390/ijerph15030536

**Published:** 2018-03-16

**Authors:** Johannes Siegrist, Jian Li

**Affiliations:** 1 Senior Professorship on Work Stress Research, Life Science Centre, University of Düsseldorf, Merowingerplatz 1a, 40225 Düsseldorf, Germany; 2 Institute of Occupational, Social and Environmental Medicine, Centre for Health and Society, Faculty of Medicine, University of Düsseldorf, Universitätsstrasse 1, 40225 Düsseldorf, Germany; jian.li@uni-duesseldorf.de

In modern societies, major changes have occurred in the world of work and employment in the recent past. A large expansion of service occupations and professions, a growing impact of information technology, digitalization and automation, and a profound impact of economic globalization are increasingly challenging traditional structures and opportunities of work and labor. With the advent of economic globalization, free market principles in conjunction with technological innovations have spread all over the world, resulting in large flows of transnational capital, trade, and labor forces. Given a growing competition and pressure toward a sizeable return on investment, a general intensification of work has been observed, along with an increase in flexible forms of employment, job insecurity, and job loss [1]. While the prevalence and impact of physically strenuous work and of exposure to traditional occupational hazards is declining, at least in modern economies, the health of working populations has been threatened by distinct stressful psychosocial work environments [2,3]. In this special issue of the International Journal of Environmental Research and Public Health, new research findings on the associations between psychosocial working conditions and the health of working people are reported, with a special focus on chronic diseases.

Research dealing with modern working conditions and health largely depends on trans-disciplinary collaborations, where knowledge from medical and basic sciences is combined with expertise from social, behavioral, and economic sciences. There is a broad consensus among the respective scientific communities that three different methodological approaches are required to critically advance the evidence base of knowledge on the health-adverse effects of work and employment. The *first approach* concerns epidemiologic investigations. Among these population-based studies, prospective cohort studies define the gold standard. This is due to their ability to distinguish between exposure and outcome in a longitudinal design, which is a prerequisite of inquiry into causal processes. Compared to cohort studies, the more frequently conducted cross-sectional epidemiologic studies suffer from a weaker scientific validity although they often provide the first opportunity of testing innovative hypotheses. Experimental studies define the *second methodological approach*. They are essential as they analyze the pathways linking occupational exposures with the development of impaired health and chronic disease. Given limited external validity, classical laboratory experiments are often supplemented by quasi-experimental and naturalistic studies, where the latter monitor psychobiological responses under conditions of everyday working life. In intervention studies, defining the *third methodological approach*, the health effects of programmed change at organizational or individual levels that aim at reducing stressful working conditions are assessed. Results of such studies are very useful, but given their methodological and practical challenges, relatively few are available so far.

This special issue offers a selection of 17 high-quality papers that cover all three methodological approaches. Six papers are based on longitudinal epidemiologic data (five prospective cohort studies and one panel study), whereas four contain data from cross-sectional investigations. One paper provides information derived from a quasi-experimental monitoring study of cardiovascular responses to stressful work environments, whereas an additional paper reviews available evidence on associations between stressful work and altered biological markers. Finally, four papers are based on intervention studies in terms of randomized control trials or less rigorously controlled changes of work-related exposures. One paper offers a narrative review of several human resource management changes in a university context. While the majority of papers were provided by researchers from Europe, two contributions come from Australia, and one each from China, South Korea, and Canada.

All longitudinal studies support the notion that stressful work environments increase the risk of poor health among exposed workers. This holds true for the risk of incident depression following exposure to unemployment and job insecurity in Germany, as demonstrated by Wege et al., for a biological risk indicator termed allostatic load among those older employed people in England who experienced chronic work stress in terms of effort–reward imbalance, as evident from the contribution of Cuiton Coronado et al., and for an elevated occurrence of circulatory disease among Australian men and women working in organizations with a low psychosocial safety climate, as documented by Becher et al. In a panel study covering data over 15 years, Sumanen et al. show that occupational class differences in sickness absence persist over time, and that male manual workers in older ages are particularly vulnerable to recurrent sickness absence. A further study from Finland, authored by Raittila et al., emphasizes that occupational class differences in physical work load among women were diminished over time only in higher occupational positions, whereas those in lower positions continued to be exposed to physical load and low job control. Conversely, high job control can buffer the effect of work load on exhaustion, as demonstrated in a small cross-lagged panel study from Germany by Konze et al. As mentioned, cross-sectional studies cannot contribute to the analysis of potentially causal processes. Yet, they can generate new hypotheses. For instance, Schou Andreassen et al. show that workaholism explains the associations of conditions of stressful work with several indicators of poor mental health among Norwegian employees, and Kim and Cho demonstrate a robust association of work–life conflicts with a prevalence of musculoskeletal disorders in a larger sample of South Korean women and men, where complaints are additionally aggravated by overtime work and high physical demands.

Whereas the paper by Borchini et al. is the only original contribution linking work stress information with physiological data, and specifically heart rate variability, a comprehensive review of available scientific evidence on associations of work stress in terms of the effort–reward imbalance model with altered biological markers is provided by Siegrist and Li. Importantly, several contributions to this issue focus on interventions and their effects on workers’ wellbeing. In their impressive investigation, Letellier et al. demonstrate that employees working in organizations that implemented health-promoting management procedures suffer substantially less from stressful work, again measured in terms of effort–reward imbalance, than those in remaining organizations. Moreover, the prevalence of psychological distress was found to be lower in the former organizations. Smaller-scale interventions concern worksite improvements that result in better mental health, as documented by Nik et al. in The Netherlands, and the beneficial effects of abdominal breathing on shoulder muscle activity, evident from the paper by Wixted et al. from Ireland. Finally, a randomized controlled trial conducted in a university setting in Denmark by Corazon et al. demonstrates that a nature-based therapy had similar favorable longer-term effects on sick leave among participants with diagnosed mental disorders as a more established cognitive behavioral therapy.

Taken together, this collection of papers offers a significant extension of current knowledge about the impact of stressful working conditions on the development of chronic diseases among working populations. The combination of three sources of evidence, epidemiological, experimental, and intervention research, must be considered as a particular strength of this special issue. It is hoped that this knowledge is used by stakeholders to implement programs and measures that aim to improve the health of working populations.
